# To Veritas

**Published:** 1808-09

**Authors:** 


### To VERITAS.

Sir,

I Hope and trust you will do me the justice to believe that I feel as anxious and as interested, as any one can do, in the discovery of the writer of the paper signed W. G. by whom, I have been grossly and scandalously misrepresented, and to whom, I replied with honest indignation. Whoever may have been the author of that offensive paper, I cannot take upon myself to say; but, although at the time I wrote my answer, I certainly had ground for suspicion, yet I feel no sort of hesitation in declaring, that *my suspicion* did *not* fall upon *Dr. Girdlestone*, and that it was *not* my intention to allude to *him* in my reply,—as I ever did consider him incapable of so unprovoked and unjust an attack upon me.

I exceedingly regret that Dr. Girdlestone's name should have been so unnecessarily and improperly, as I conceive, brought forward on such an occasion, though, at the same time, it affords me great pleasure in embracing this opportunity of doing justice to his character; for which, as that of a gentleman and a scholar, I feel the highest esteem.

HARLESTONENSIS.

